# Deficiency of Stabilin-1 in the Context of Hepatic Melanoma Metastasis

**DOI:** 10.3390/cancers16020441

**Published:** 2024-01-19

**Authors:** Sebastian A. Wohlfeil, Ana Olsavszky, Anna Lena Irkens, Verena Häfele, Bianca Dietsch, Niklas Straub, Sergij Goerdt, Cyrill Géraud

**Affiliations:** 1 Department of Dermatology, Venereology, and Allergology, University Medical Center and Medical Faculty Mannheim, Heidelberg University, and Center of Excellence in Dermatology, 68135 Mannheim, Germanyanna.irkens@medma.uni-heidelberg.de (A.L.I.); verena.haefele@web.de (V.H.); bianca.dietsch@medma.uni-heidelberg.de (B.D.); niklas.straub@medma.uni-heidelberg.de (N.S.); sergij.goerdt@umm.de (S.G.); cyrill.geraud@umm.de (C.G.); 2 Section of Clinical and Molecular Dermatology, Medical Faculty Mannheim, Heidelberg University, 68135 Mannheim, Germany; 3 Skin Cancer Unit, German Cancer Research Center (DKFZ), 69120 Heidelberg, Germany; 4 European Center for Angioscience, Medical Faculty Mannheim, Heidelberg University, 68135 Mannheim, Germany

**Keywords:** cutaneous melanoma, melanoma metastasis, liver metastasis, Stablin-1, melanoma therapy

## Abstract

**Simple Summary:**

Deficiency and targeting of Stabilin-1, which is expressed by liver sinusoidal endothelial cells and subsets of macrophages, has been shown to improve anti-tumor immune responses in preclinical and early clinical studies. This study investigated whether targeting of Stab1 might also influence melanoma liver colonization, as hepatic metastasis of melanoma is a poor prognostic factor. In two models of hepatic melanoma metastasis, no direct influence of anti-Stab1 treatment was found. However, effects on the local microenvironment were observed. We suggest that the organ- and tumor-specific effects of anti-Stab1 therapies should be further considered and analyzed in future human studies.

**Abstract:**

Background: This study analyzed the role of Stabilin-1 on hepatic melanoma metastasis in preclinical mouse models. Methods: In *Stabilin-1^−/−^* mice (Stab1 KO), liver colonization of B16F10 *luc2* and Wt31 melanoma was investigated. The numbers, morphology, and vascularization of hepatic metastases and the hepatic microenvironment were analyzed by immunofluorescence. Results: While hepatic metastasis of B16F10 *luc2* or Wt31 melanoma was unaltered between Stab1 KO and wildtype (Ctrl) mice, metastases of B16F10 *luc2* tended to be smaller in Stab1 KO. The endothelial differentiation of both types of liver metastases was similar in Stab1 KO and Ctrl. No differences in initial tumor cell adhesion and retention to the liver vasculature were detected in the B16F10 *luc2* model. Analysis of the immune microenvironment revealed a trend towards higher levels of CD45^+^Gr-1^+^ cells in Stab1 KO as compared to Ctrl in the B16F10 *luc2* model. Interestingly, significantly higher levels of POSTN were found in the matrix of hepatic metastases of Wt31, while liver metastases of B16F10 *luc2* showed a trend towards increased deposition of RELN. Conclusions: Hepatic melanoma metastases show resistance to Stabilin-1 targeting approaches. This suggests that anti-Stab1 therapies should be considered with respect to the tumor entity or target organs.

## 1. Introduction

Stabilin-1 (Stab1), also referred to as Clever-1 or Feel-1, is a scavenger receptor expressed by subsets of macrophages and sinusoidal endothelial cells in the liver, spleen, lymph nodes, and bone marrow [1,2,3]. As a type I transmembrane protein, it consists of several EGF-like domains, seven fasciclin-1 domains, a nonfunctional hyaluronic acid-binding X-link domain, a transmembrane, and a short cytoplasmic domain. Despite close homology to Stabilin-2 (Stab2), these class H scavenger receptors differ functionally.

Ligands of Stab1 include SPARC, Stab1 interacting chitinase-like protein (SI-CLP) [4], placental lactogen [5], oxidized and acetylated low-density lipoprotein (oxLDL) [6,7], advanced glycation end products [8], or GDF-15 [9]. Recently, transforming growth factor-β-induced protein (TGFBI), Periostin (POSTN), and Reelin (RELN) have been identified as novel ligands of Stab1 and Stab2 [10,11]. Hepatic clearance functions of Stab1 indirectly influence the progression of inflammatory diseases. Atherosclerosis is improved by targeting Stab1 or Stab2, which leads to an anti-inflammatory switch in the plasma proteome and decreases aortic plaque formation in a mouse model [10]. Further, a double knock-out (KO) of Stab1 and Stab2 results in a perisinusoidal fibrosis in the liver and a glomerulosclerosis of the kidneys [12]. Interestingly, Stab1 KO only present with a mild perisinusoidal fibrosis and no glomerulofibrotic nephropathy, which suggests a functional compensation of the loss of Stab1 by Stab2. Last, ligands of Stab1 also influence cancer progression as SPARC inhibits metastasis of breast cancer cells [13] or reduces the growth of neuroblastoma [14], Lewis lung carcinoma, or EL4 cells [15].

The functions of Stab1 extend beyond its role as a scavenging receptor. Leukocyte migration to draining lymph nodes and the spleen is controlled by Stab1 [16,17]. Even in cancer development, this is relevant, as Stab1 recruits both immunosuppressive macrophages and regulatory T cells (Treg) [18]. In addition, growth and lymph node metastasis of subcutaneous B16F10 melanomas are significantly reduced by genetic deficiency of *Stab1* or targeting with an anti-Stab1 antibody in a mouse model [18]. In patients with advanced colorectal carcinoma (CRC) or gastric cancer, higher levels of Stab1-positive macrophages correlated with decreased tumor-specific survival [19,20]. Since these studies indicated an immunosuppressive effect of Stab1 on the tumor microenvironment, targeting of Stab1 was further investigated. In the absence of *Stab1*, macrophages polarize into a more inflammatory phenotype. The combination of anti-Stab1 and anti-PD1 treatments leads to increased levels of CD8^+^ T cells in murine tumors, with no additive effect on tumor reduction. However, reprogramming of the immune microenvironment might be especially important in patients with resistance to immune checkpoint inhibition. As early data of the phase I/II MATINS trial (NCT03733990) with the humanized anti-Stab1 antibody bexmarilimab indicate, a proinflammatory switch in monocytes is also induced in patients [21]. Currently, safety signals and efficacy are under further investigation.

In this study, we investigated whether the targeting of Stab1 might also influence melanoma liver colonization, as hepatic metastasis of melanoma is well described as a poor prognostic factor [22,23,24]. To this end, mice with a conditional KO of *Stab1* were used and multiple aspects during melanoma liver colonization were analyzed in detail.

## 2. Materials and Methods

### 2.1. Animals

For in vivo experiments, Stab1 KO (B6.129S2-Stab1^tm1.1Cger 16^) [12] were used. Homozygous littermates or purchased C57Bl/6J wildtype (Janvier Labs, Le Genest-Saint-Isle, France) served as Ctrl. Heterozygous mice were not used. All animals were hosted in single ventilated cages (Sealsafe plus DGM™, Techniplast, Buguggiate, Italy; Bedding H0234-20, Ssniff, Soest, Germany) in a 12 h/12 h day/night cycle under specific-pathogen-free conditions and fed ad libitum with a standard rodent diet (ssniff^®®^R/M-H autoclavable, V1534-000, Ssniff, Soest, Germany). For DNA extraction and genotyping, the KAPA HotStart Mouse Genotyping Kit (KK7352, Merck, Darmstadt, Germany) and primers (Metabion international AG, Planegg/Steinkirchen, Germany) were utilized (Supplementary Table S1).

### 2.2. Cell Lines

B16F10 *luc2* and Wt31 mouse melanoma cells were used for in vivo experiments. B16F10 *luc2* cells were bought from ATCC (Manassas, VA, USA). The murine melanoma cell line Wt31 was derived from *Tyr::NrasQ61K/°; INK4a^−/−^* mice [25] and was a generous gift from O. Sansom (Beatson Institute for Cancer Research, Scotland). They were maintained in RPMI 1640 media (Thermo Fisher Scientific, Waltham, MA, USA) with 10% (*v*/*v*) fetal calf serum (FCS) and 100 U/mL penicillin/streptomycin at 37 °C, 5% CO_2_. For all in vivo experiments, the same passages of B16F10 *luc2* or Wt31 were used. After the thawing of the cells, they were not passaged more than three times, and the maximum culture time prior to in vivo experiments was one week. For cell authentication STR sequencing was performed (Eurofins, Ebersberg, Germany) and confirmed unique profiles of all used cell lines. In addition, cells were distinguished by pigmentation status, morphology, or bioluminescence.

### 2.3. Liver and Lung Colonization Assay

Liver and lung colonization assays were performed as previously established and described [26,27]. Female, age-matched mice between 10 and 12 weeks were used for in vivo experiments. To study liver colonization, intrasplenic or intravenous injections of tumor cells were performed. After spleen injection of 1.5 × 10^5^ B16F10 *luc2* cells, the mice were sacrificed after 14 days, and bioluminescence imaging (BLI) measurements of the livers and lungs were performed. 2.5 × 10^6^ Wt31 melanoma cells were applied by tail vein injection, and mice were sacrificed after 19 days. To analyze the retention of melanoma cells in the liver 3 × 10^5^ B16F10 *luc2* cells were injected intrasplenically (i.s.) and analysis was performed 90 min after intrasplenic injection by BLI.

### 2.4. BLI

An IVIS^®^ Lumina LT In Vivo Imaging System (Caliper Life Sciences, Perkin Elmer, Waltham, MA, USA) was used. Ten minutes after intraperitoneal application of luciferin (D-luciferin 1-(4,5-dimethoxy-2-nitrophenyl)ethyl ester, 7903, 30 mg/mL, BioVision, Milpitas, CA, USA), mice were sacrificed, livers were removed, put into a Petri dish (664160, Greiner Bio One, Kremsmünster, Austria), and were imaged ex vivo with the IVIS^®^ Lumina LT with an exposure time of 45 s.

### 2.5. Liver Dissection, Cryopreservation, and Paraffin Embedding

Mice were sacrificed by cervical dislocation. Livers were either fixed in 4% PFA at 4 °C for 24 h to 72 h, followed by paraffin embedding according to standard protocols, or livers were embedded in OCT (Sakura Finetek Europe B.V. KvK, Alphen Aan Den Rijn, The Netherlands).

### 2.6. Immunofluorescences and Routine Histology

Paraffin-embedded tissue was cut into 3 µm thick sections. Then routine stainings hematoxylin & eosin (H&E), Sirius red (SR), periodic acid-Schiff (PAS), and Prussian blue (Fe) were performed according to standard protocols of the manufacturer. Immunofluorescence stainings were performed on cryoconserved tissue, which was cut into 8 µm thick slices and was air dried. For fixation, 4% paraformaldehyde (PFA) (0335, Carl Roth, Karlsruhe, Germany) was put on the tissue for ten minutes. Afterwards, PBS with 5% normal donkey serum (017-000-121, Dianova, Hamburg, Germany) was placed on the tissue for blocking for 30 min.

### 2.7. Antibodies

Primary antibodies: rat anti-CD31 (DM3614P, Dianova, Hamburg, Germany), goat anti-CD32b (AF1460, R&D Systems, Minneapolis, MN, USA), goat anti-Lama4 (AF3837, R&D Systems, Minneapolis, MN, USA), rabbit anti-Desmin (ab32362, Abcam, Cambridge, UK), rabbit anti-CD3 (100202, Biolegend, San Diego, CA, USA), rat anti-CD4 (100402, Biolegend, San Diego, CA, USA), rat anti-CD8a (100802, Biolegend, San Diego, CA, USA), rabbit anti-F4/80 (30325S, Cell Signaling, Danvers, MA, USA), rat anti-CD11b (101202, Biolegend, San Diego, CA, USA), rabbit anti-CD11c (97585S, Cell Signaling, Danvers, MA, USA), rat anti-MHCII (14-5321-85, eBioscience, Thermo Fisher Scientific, Waltham, MA, USA), rabbit anti-FoxP3 (12653, Cell Signaling, Danvers, MA, USA), rabbit anti-CD45 (ab10558, abcam, Cambridge, UK), rat anti-Ly6C (ab15627, abcam, Cambridge, UK), rat anti-Gr1 (ab 25377, abcam, Cambridge, UK), goat anti-Reelin (AF3820, R&D Systems, Minneapolis, MN, USA), goat anti-Periostin (AF2955, R&D Systems, Minneapolis, MN, USA), rabbit anti-TGFBI (ab170874, abcam, Cambridge, UK). Secondary antibodies: donkey Alexa-Fluor 488, Alexa-Fluor 647, and Cy3-conjugated secondary antibodies were purchased from Dianova (Hamburg, Germany).

### 2.8. Image Acquisition and Processing

Pictures of routine histology or immunofluorescences were acquired by an Eclipse Ni-E motorized upright microscope (Nikon Instruments Europe BV, Amsterdam, The Netherlands) using 10×, 20× or 40× CFI Plan Apochromat Lambda series objective lenses, an Intensilight Epifluorescence Illuminator, a DS-Ri2 high-definition color camera, and a DS-Qi2 high-definition monochrome camera. The system was controlled by NIS-Elements AR 5.02 software (Nikon Instruments, Tokyo, Japan). During acquisition, data were not compressed. Fluorescence images were acquired as z-stacks.

Image processing included background reduction, deconvolution, and extended depth of focus using NIS-Elements AR 5.02 and ImageJ software, version 1.54f (NIH, USA). Ligand depositions were analyzed by Image J. To define intratumoral and peritumoral areas, regions of interest (ROI) were defined. The percentage of ligand deposition in relation to the ROI in the respective area was determined. Means for each mouse were calculated and statistically compared. Immunofluorescence stainings of immune cells were scanned with an automated slide scanner Axio Scan.Z1 (Zeiss, Jena, Germany). Whole liver sections were analyzed by Imaris 9.9 (Oxford Instruments, Abingdon, UK). Briefly, the whole liver tissue was analyzed as DAPI positive area by calculating a surface. Double positive immune cells were detected by a threshold-based spot calculation. Thresholds were set for two immune cell markers and the DAPI signal. The number of positive spots was counted in the total hepatic tissue, metastatic tissue was excluded from the analysis.

### 2.9. Statistical Analysis

All statistical analyses and graphical displays were performed with GraphPad Prism 9 (Graph Pad, La Jolla, CA, USA) and mean ± SEM is presented. For statistical analysis, an unpaired, two-tailed *t*-test was applied if data met the criteria of normality. Otherwise, a Mann–Whitney test was used. Differences between data sets with *p* < 0.05 were considered statistically significant.

## 3. Results

### 3.1. Influence of Stab1 on Melanoma Liver Colonization

**Figure 1:**
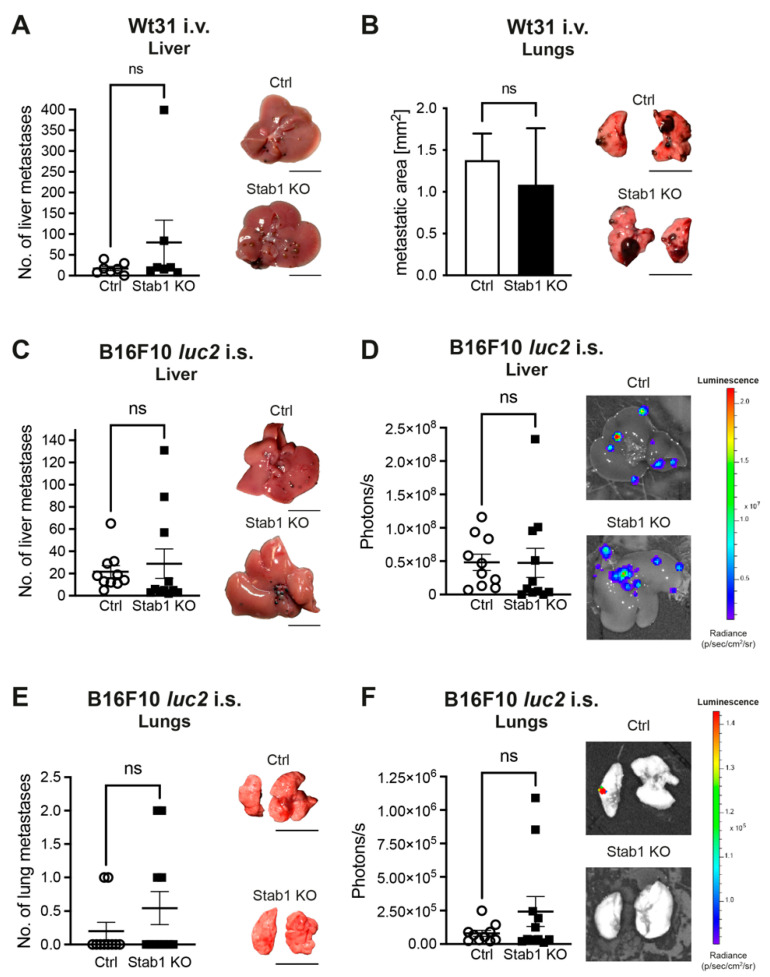
Deficiency of *Stab1* does not affect liver colonization by Wt31 or B16F10 *luc2* melanoma. (**A**) The numbers of macroscopic liver metastases in Ctrl or Stab1 KO after i.v. injection of 2.5 × 10^6^ Wt31 melanoma cells are compared (*p* = 0.4021, Mann–Whitney test; n = 7/group). Mice were sacrificed on day 19. The experiment was repeated three times. Representative images of livers with Wt31 metastases are shown. Circles illustrate the numbers of Wt31 liver metastases of individual Ctrl mice, whereas squares are used for Stab1 KO. Scale bars = 1 cm. (**B**) The macroscopic areas of lung metastases in Ctrl or Stab1 KO after i.v. injection of 2.5 × 10^6^ Wt31 melanoma cells are compared (*p* = 0.1049, Mann–Whitney test; n = 7/group). Mice were sacrificed on day 19. The experiment was repeated three times. Representative images of lungs with Wt31 metastases are shown. Scale bars = 1 cm. (**C**) The numbers of liver metastases of B16F10 *luc2* melanoma after intrasplenic (i.s.) injection are presented (*p* = 0.1782, Mann–Whitney test). 1.5 × 10^5^ B16F10 *luc2* cells were injected into Ctrl (n = 10) or Stab1 KO (n = 11). The experiment was repeated four times. Representative images of colonized livers are shown. Circles illustrate the numbers of B16F10 *luc2* liver metastases of individual Ctrl mice, whereas squares are used for Stab1 KO. Scale bars = 1 cm. (**D**) Ex vivo BLI images of livers with metastases of B16F10 *luc2* in Ctrl and Stab1 KO. Quantification of livers determined as region of interest on day14 after intrasplenic injection of B16F10 *luc2* melanoma cells (*p* = 0.2512, Mann–Whitney test). Color scale: min = 1.57 × 10^6^ p/s/cm^2^/sr; max = 2.13 × 10^7^ p/s/cm^2^/sr. Circles illustrate data points of individual Ctrl mice, whereas squares are used for Stab1 KO. (**E**) Macroscopic visible lung metastases were quantified. The numbers of macroscopic lung metastases of B16F10 *luc2* in Ctrl or Stab1 KO are presented (*p* = 0.4567, Mann–Whitney test). A pooled analysis of four different experiments is presented. Circles illustrate data points of individual Ctrl mice, whereas squares are used for Stab1 KO. Representative images of colonized lungs are shown. Scale bars = 1 cm. (**F**) Ex vivo BLI images of lungs with metastases of B16F10 *luc2* in Ctrl and Stab1 KO. Quantification of lungs determined as region of interest on day14 after intrasplenic injection of B16F10 *luc2* melanoma cells (*p* = 0.7045, Mann–Whitney test). Color scale: min = 9.04 × 10^4^ p/s/cm^2^/sr; max = 1.43 × 10^5^ p/s/cm^2^/sr. Data information: ns = not significant. Circles illustrate data points of individual Ctrl mice, whereas squares are used for Stab1 KO.

As Stab1 is being investigated as a target in multiple different cancers, such as melanoma, we decided to investigate its impact on hepatic melanoma metastasis. This is relevant as liver metastases mediate resistances in approved melanoma therapies [22,23,24]. To this end, mice with a constitutive KO of *Stab1* were used [12]. The number of liver metastases was similar between Stab1 KO and Ctrl after i.v. injection of Wt31 melanoma cells (Figure 1A). In addition, lung colonization did not differ between the two groups (Figure 1B).

The body weight of Stab1 KO was significantly increased after i.v. injection of Wt31 (*p* = 0.0484), while the weight of the livers and lungs were unaltered (Supplementary Materials Figure S1A–C). In a second model, B16F10 *luc2* melanoma cells were injected into the spleen of Stab1 KO and Ctrl. On day 14, no difference in the number of liver metastases was found (Figure 1C), which was also confirmed by ex vivo BLI (Figure 1D). The lungs as secondary metastatic site also exhibits no difference in the number of metastases between Stab1 KO and Ctrl (Figure 1E,F). In line with that, body weights and weights of livers and lungs were unaltered between Stab1 KO and Ctrl (Supplementary Materials Figure S1D–F).

### 3.2. Initial Tumor Cell Adhesion and Retention

**Figure 2:**
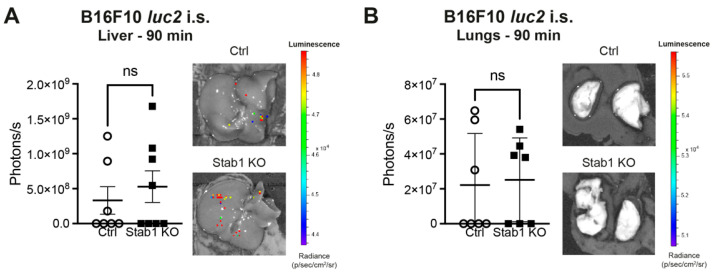
Initial adhesion and retention of B16F10 *luc2* in the liver is not altered in Stab1 KO. (**A**) Ex vivo BLI images of livers with metastases of B16F10 *luc2* in Ctrl and Stab1 KO. Quantification of livers determined as a region of interest 90 min after intrasplenic injection of B16F10 *luc2* melanoma cells (*p* = 0.8665, Mann–Whitney test). The experiment was repeated three times. Color scale: min = 4.38 × 10^4^ p/s/cm^2^/sr; max = 4.86 × 10^4^ p/s/cm^2^/sr. Circles illustrate data points of individual Ctrl mice, whereas squares are used for Stab1 KO. (**B**) Ex vivo BLI images of lungs with metastases of B16F10 *luc2* in Ctrl and Stab1 KO. Quantification of lungs determined as a region of interest 90 min after intrasplenic injection of B16F10 *luc2* melanoma cells (*p* = 0.7104, Mann–Whitney test). The experiment was repeated three times. Color scale: min = 5.01 × 10^4^ p/s/cm^2^/sr; max = 5.56 × 10^4^ p/s/cm^2^/sr. Circles illustrate data points of individual Ctrl mice, whereas squares are used for Stab1 KO. Data information: ns = not significant.

Since Stab1 mediates the adhesion of multiple cell types and even promotes lymph node metastasis of melanoma [16,17,18], initial tumor cell adhesion and retention to the liver were studied by BLI. Similar luminescence intensities were detected 90 min after i.s. injection of B16F10 *luc2* both in the livers (Figure 2A) and lungs (Figure 2B) of Stab1 KO and Ctrl. This indicates that Stab1 does not impair or mediate tumor cell adhesion and retention to the hepatic sinusoids in this melanoma model.

### 3.3. Characterization of Liver Tissue and Melanoma Metastases

**Figure 3:**
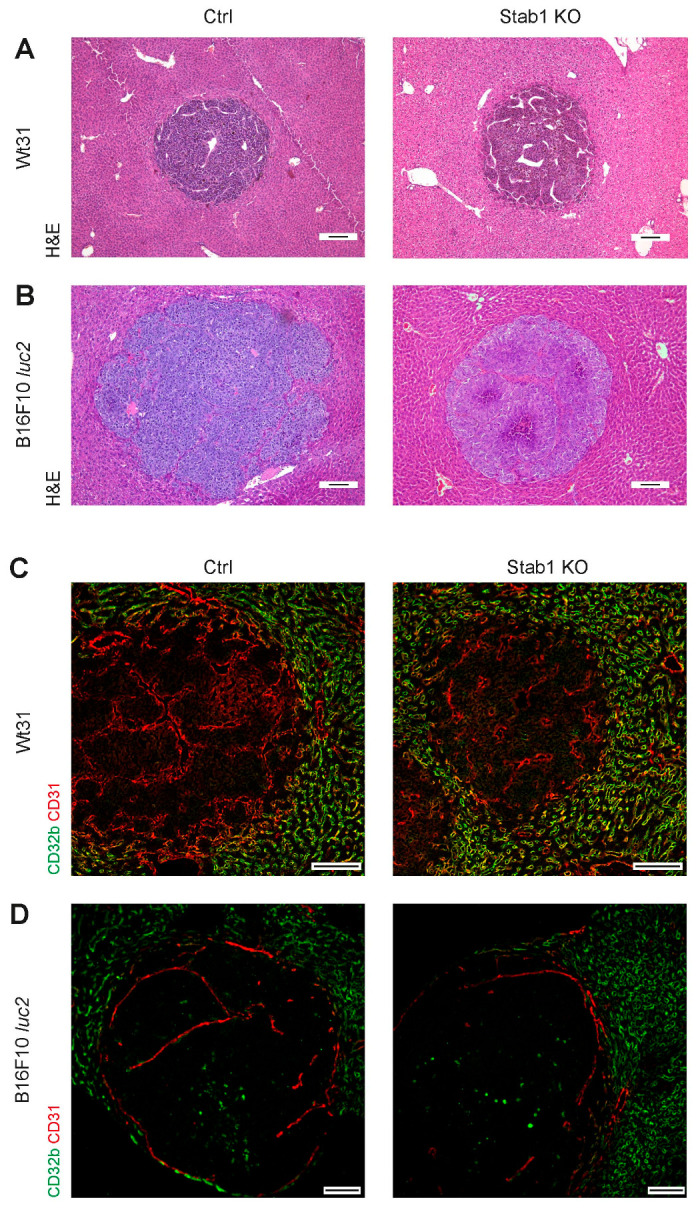
Morphology and vascularization of hepatic metastases of Wt31 or B16F10 luc2 melanoma. (**A**,**B**) Images of H&E stainings of hepatic metastases of Wt31 (**A**) or B16F10 luc2 melanoma (**B**) in Ctrl or Stab1 KO livers. Scale bars: 100 µm. (**C**,**D**) Immunofluorescences of hepatic metastases of Wt31 (**C**) or B16F10 luc2 melanoma (**D**) for CD31 and CD32b in Ctrl and Stab1 KO. Scale bars: 100 µm.

Deficiency of Stab1 on hepatic endothelial cells could affect the pattern of hepatic melanoma metastasis or the development and maturation of its vasculatures. Therefore, the morphology and size of melanoma liver metastases were studied. Hepatic metastases of both Wt31 and B16F10 *luc2* melanoma showed a pushing type histopathological growth pattern in Stab1 KO and Ctrl (Figure 3A,B). Peritumoral liver tissues of Stab1 KO or Ctrl mice that were injected with Wt31 i.v. or B16F10 *luc2* i.s. were both not altered morphologically (Supplementary Materials Figures S2 and S3). A common perisinusoidal fibrosis was found in Stab1 KO. Notably, in the melanoma metastases themselves, no increased collagen deposition was detectable by SR staining (Supplementary Materials, Figure S4A,B). Interestingly, a trend towards smaller metastases of B16F10 *luc2* melanoma in Stab1 KO in relation to Ctrl was found (*p* = 0.0635), while no difference in size between both groups was detected for Wt31 metastases (Supplementary Materials, Figure S4C,D). However, the pattern of vascular differentiation of Wt31 or B16F10 *luc2* melanoma metastases was similar between Stab1 and Ctrl. Intratumoral blood vessels showed expression of CD31, a typical continuous endothelial cell marker (Figure 3C,D; Supplementary Materials, Figure S5). Likewise, the intratumoral expression of laminin subunit alpha 4 (LAMA4), a compound of the endothelial basement membrane of continuous endothelial cells, was similar between both groups (Supplementary Materials, Figure S5C,D). Furthermore, hepatic stellate cell activation appeared to be the same between Stab1 KO and Ctrl, as expression of DESMIN was not affected by deficiency of Stab1.

### 3.4. Analysis of Hepatic Immune Microenvironment

**Figure 4:**
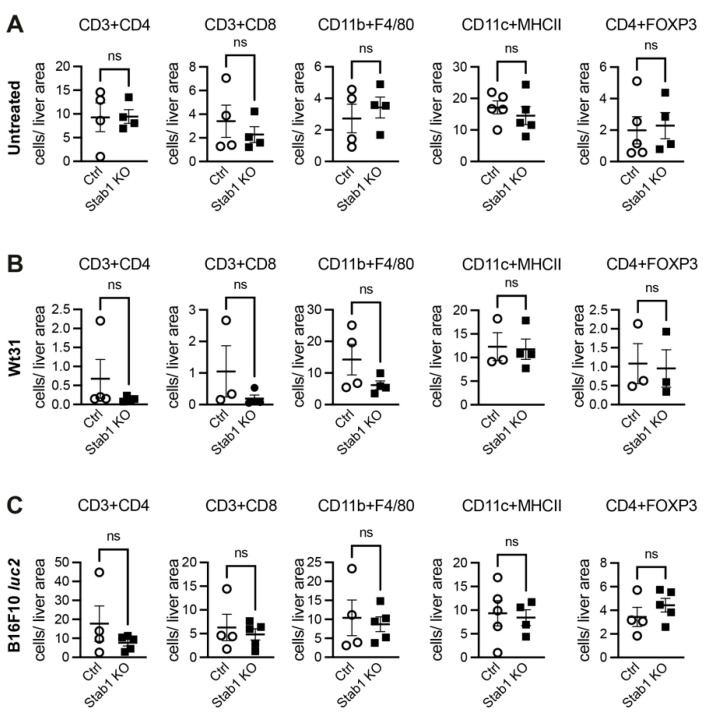
Analysis of the immune cell composition in livers of Ctrl and Stab1 KO without metastases or with melanoma metastases of B16F10 *luc2* or Wt31. Livers of Ctrl or Stab1 KO without metastases (**A**) or with metastases of Wt31 (**B**) or B16F10 *luc2* (**C**) were analyzed for T cells (CD3+CD4, CD3+CD8), macrophages (CD11b+F4/80), dendritic cells (CD11c+MHCII), or regulatory T cells (CD4+FOXP3) by immunofluorescence. By whole slide imaging, the numbers of respective immune cells were objectively quantified. The numbers of cells were set in relation to a liver area of 1 × 10^6^ µm^2^. Metastases were excluded from the analysis. (**A**) Untreated livers of Ctrl or Stab1 KO were analyzed. CD3+CD4: *p* = 0.8857; CD3+CD8: *p* = 0.8857; CD11b+F4/80: *p* = 0.6857; CD11b+MHCII: *p* = 0.4206; CD4+FOXP3: *p* = 0.7302; (**B**) Livers of Ctrl or Stab1 KO with Wt31 melanoma metastases were analyzed. CD3+CD4: *p* = 0.2000; CD3+CD8: *P* = 0.2286; CD11b+ F4/80: *p* = 0.2000; CD11c + MHCII: *p* ≥ 0.9999; CD4+FOXP3: *p* = 0.7000; (**C**) Livers of Ctrl or Stab1 KO T cells with B16F10 *luc2* melanoma metastases were analyzed. CD3+CD4: *p* = 0.5556; CD3+CD8: *p* > 0.9999; CD11b+F4/80: *p* > 0.9999; CD11c+MHCII: *p* = 0.9048; CD4+FOXP3: *p* = 0.4127. Data information: ns = not significant.

As Stab1 is expressed on subsets of macrophages, its deficiency could have an impact on the hepatic immune microenvironment itself. Therefore, hepatic immune subsets were analyzed in detail by immunofluorescences. In untreated, healthy mice, the same levels of CD3^+^CD4^+^ or CD3^+^CD8^+^ T cells, CD11b^+^F4/80^+^ hepatic macrophages, CD11c^+^MHCII^+^ dendritic cells, CD4^+^FOXP3^+^ regulatory T cells, CD45^+^Gr-1^+^ myeloid cells, or CD45^+^Ly6C^+^ inflammatory monocytes were detected (Figure 4A; Supplementary Materials Figure S6A). Upon liver colonization with Wt31 melanoma, the levels of both CD4^+^ and CD8^+^ decreased in the liver, but no statistical differences between Stab1 KO and Ctrl were detected (Figure 4B; Supplementary Materials Figure S6B). Interestingly, liver colonization with B16F10 *luc2* tended to increase levels of CD45^+^ Gr-1^+^ myeloid cells in hepatic tissue of Stab1 KO as compared to Ctrl (*p* = 0.0571; Figure 4C; Supplementary Materials Figure S6C).

### 3.5. Deposition of Stabilin Ligands

**Figure 5:**
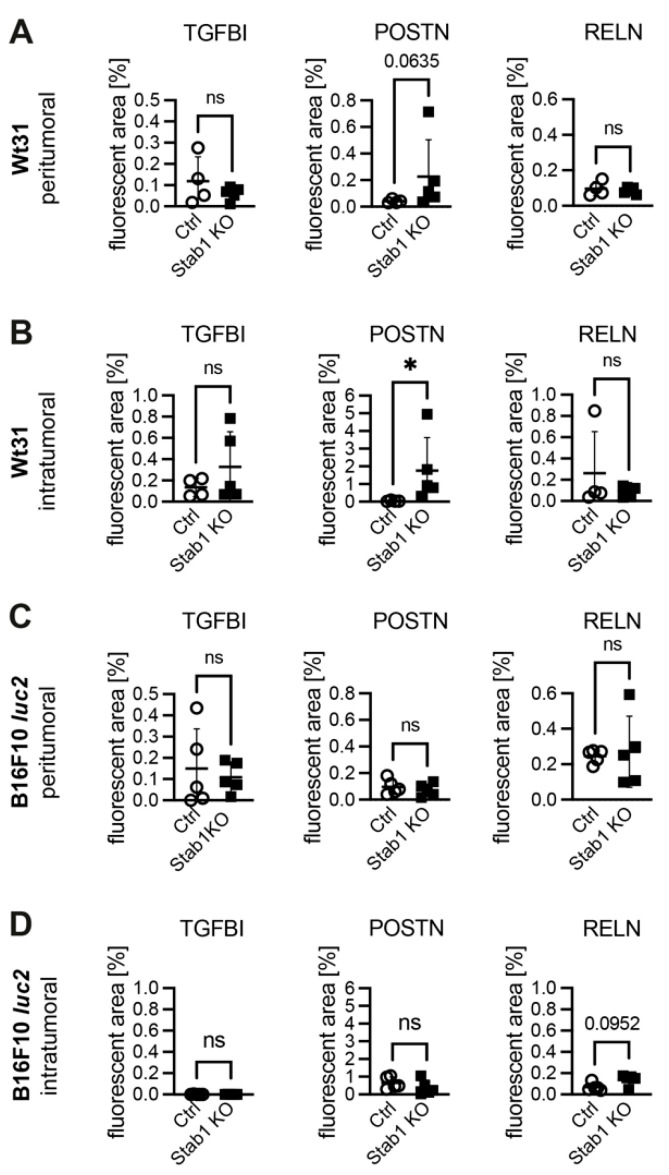
Deposition of ligands of Stab1 was determined in tumor-occupied livers of Ctrl and Stab1 KO. Immunofluorescences for TGFBI, POSTN, or RELN, ligands of Stab1, were quantified. The fluorescent area per 100.000 µm^2^ tissue [%], either peritumoral or intratumoral, was calculated and compared between the groups. (**A**) Graphs show the comparison of the deposition of TGFBI, POSTN, and RELN in peritumoral liver tissue of Ctrl and Stab1 KO that received Wt31 i.v. TGFBI: *p* = 0.5952; POSTN: *p* = 0.0635; RELN: *p* = 0.9603. (**B**) Graphs show the comparison of the deposition of TGFBI, POSTN, and RELN in Wt31 melanoma liver metastases of Ctrl and Stab1 KO. TGFBI: *p* = 0.4127; POSTN: *p* = 0.0159; RELN: *p* > 0.9999. (**C**) Graphs show the comparison of the deposition of TGFBI, POSTN, and RELN in peritumoral liver tissue of Ctrl and Stab1 KO that received B16F10 *luc2* i.s. TGFBI: *p* = 0.8413; POSTN: *p* = 0.6905; RELN: *p* > 0.9999. (**D**) Graphs show the comparison of the deposition of TGFBI, POSTN, and RELN in B16F10 *luc2* melanoma metastases of Ctrl and Stab1 KO. TGFBI: *p* = 0.8413; POSTN: *p* = 0.4206; RELN: *p* = 0.0952. Data information: * *p* < 0.05; ns = not significant.

Last, we investigated whether local or systemic clearance functions of Stab1 may affect the local deposition of Stabilin ligands in the extracellular matrix of hepatic metastases. To this end, the depositions of TGFBI, POSTN, and RELN were analyzed. In untreated, healthy livers, significantly higher levels of TGFBI (*p* = 0.0159) and POSTN (*p* = 0.0079) were found, while the deposition of RELN was similar (Supplementary Materials Figure S7). In mice injected with Wt31 i.v., a trend towards a higher level of POSTN in Stab1 KO as compared to Ctrl was detected in peritumoral areas (*p* = 0.0635), while no differences were seen for TGFBI or RELN (Figure 5A; Supplementary Materials Figure S8A). Interestingly, this deposition of POSTN in Stab1 KO increased almost by 8-fold in Wt31 metastases and was significantly higher in relation to Ctrl (*p* = 0.0159; Figure 5B; Supplementary Materials Figure S8B). Hepatic metastases of B16F10 *luc2* altered neither the deposition of TGFBI, POSTN, nor RELN in peritumoral areas (Figure 5C; Supplementary Materials Figure S9A). However, in B16F10 *luc2* metastases themselves a trend towards increased levels of RELN (*p* = 0.0952) was observed (Figure 5D; Supplementary Materials Figure S9B). Notably, there was almost no expression of TGFBI in metastases of B16F10 *luc2*.

Altogether, the establishment and progression of melanoma liver colonization were only mildly altered by *Stab1* deficiency, as liver metastases of B16F10 *luc2* tended to be smaller in Stab1 KO. In addition, alterations of the local immune microenvironment and ligand depositions were seen in hepatic metastases of Stab1 KO.

## 4. Discussion

Challenges of novel anti-cancer therapies include resistance to immune checkpoint inhibition (ICI). Regarding melanoma, liver metastases are poor prognostic factors for ICI [22,23]. At the hepatic site, monocyte-derived macrophages induce apoptosis of tumor-specific CD8+ T cells, resulting in depletion from the peripheral circulation, leading to local and systemic resistance to ICI [23]. Therefore, macrophages are being discussed as novel targets to overcome therapy resistance [28]. One therapeutic approach directs against the scavenger receptor Stab1 [28], with a cell-specific expression only on subsets of macrophages and sinusoidal endothelial cells [1,2,3].

The influence of Stab1 on tumor development has already been studied in several tumor models. The growth of subcutaneous B16 melanomas is significantly reduced by a global knockout (KO) of Stab1 [18]. As treatment with anti-Stab1 antibodies also decreases both the growth and metastasis of B16 melanoma and EL-4 lymphoma, this strategy might be used therapeutically [18]. Further, a full KO of *Stab1* also significantly decreases the growth of murine mammary adenocarcinoma [29]. In contrast, loss of *Stab1* did not result in altered melanoma liver colonization in our study, as the numbers of liver metastases were similar in Stab1 KO and Ctrl after i.v. injection of Wt31 or spleen injection of B16F10 *luc2*. However, a tendency towards smaller metastases of B16F10 *luc2* (*p* = 0.0635), but not Wt31 melanoma, was observed in Stab1 KO as compared to Ctrl. This suggests that the effects of a modulation of Stab1 depend on the tumor entity. Data from the phase I/II MATINS trial with Bexmarilimab, a human anti-Stab1 antibody, also indicate such notion [30]. However, as the first-in-human study, this trial was designed on drug safety and tolerability, so that conclusions on tumor responses need to be verified in larger patient cohorts and phase III studies. As initial tumor cell adhesion and retention of B16F10 *luc2* after spleen injection were not affected by deficiency of *Stab1*, the influence of Stab1 in the B16F10 *luc2* model seems to be restricted to later phases of liver colonization. Moreover, our findings underline organ-specific difficulties in targeting liver metastases. We hypothesize that effects might be limited by the restricted expression of Stab1 in the liver, as it is mainly expressed on liver sinusoidal endothelial cells (LSEC). This is supported by a study in human breast cancer patients that demonstrated only a very mild infiltration by STAB1^+^ cells in the liver [31]. Further, the levels of STAB1^+^ cells were unaltered by liver metastasis, while the density of intratumoral CD3^+^ T cells and M1-like macrophages significantly decreased [31]. Interestingly, previous studies could not clearly distinguish the effects of Stab1 between endothelial cells and macrophages, as only global Stab1 KO mice showed statistically significant effects on tumor growth reduction as compared to endothelial or macrophage-specific KO mice [18]. Last, we suggest that effects of a deficiency of *Stab1* in our model could be also masked by the highly tolerogenic immune milieu of the liver [32]. Altogether, this indicates that modulation of Stab1 might strongly depend on tumor- and organ-specific factors.

Detailed analyses of the hepatic microenvironment after melanoma colonization revealed an altered deposition of Stabilin ligands which could also mask potential beneficial effects of targeting Stab1. Healthy, untreated mice showed significantly higher levels of TGFBI (*p* = 0.0159) and POSTN (*p* = 0.0079) upon loss of *Stab1* as compared to Ctrl. In disease processes such as liver fibrosis or arteriosclerosis, reduced clearance functions by loss of *Stab1* are further exaggerated [10,33]. Levels of TGFBI in the liver correlate with the intensity of perisinusoidal fibrosis in Stab1 KO [33]. In cancer, stromal expression of POSTN is a predictor of poorer survival in CRC [34]. In tumor bearing, *Stab1* deficient mice an accumulation of POSTN was predominantly seen in the matrix of Wt31 melanoma metastases (*p* = 0.0159). In hepatocellular carcinoma, POSTN is associated with reduced survival as it promotes migration and invasion of tumor cells [35]. Further, POSTN signaling via the AKT and YAP pathways increases metastasis of gastric cancer [36]. In the intratumoral stroma of B16F10 *luc2* liver metastases, a tendency towards higher levels of RELN in Stab1 KO as compared to Ctrl (*p* = 0.0952) and almost a complete loss of TGFBI in both Stab1 KO and Ctrl was observed. In hepatocellular carcinoma, low levels of RELN correlate with increased recurrence [37]. Further, TGFBI has a pro-tumorigenic function for hepatic metastasis of CRC and strongly regulates angiogenesis [38]. Wt31 melanoma shows a higher efficacy in colonizing the liver as compared to B16F10 *luc2* [27]. The distinct stromal ligand deposition between these two melanoma models might contribute to differences between B16F10 *luc2* and Wt31 metastases, such as the tendency towards smaller metastases of B16F10 *luc2*.

Recent findings highlight the important clearance functions of the scavenger receptors Stab1 or Stab2 since rare protein-coding variants in *STAB1* or *STAB2* were mainly associated with alterations of plasma protein levels in a large cohort of UK patients [11]. To better understand therapies against scavenging receptors, such as Stab1, corresponding ligand repertoires should be further investigated. Despite no significant influence on hepatic metastasis, therapies directed against Stab1 might lead to changes in the hepatic microenvironment and plasma proteome with potential secondary beneficial effects. Murine models suggest that the response to anti-Stab1 treatment is tumor- and organ-specific. To follow up on this, future studies in patients with Bexmarilimab are highly anticipated.

## 5. Conclusions

Despite promising results of anti-Stab1 treatments in different tumors and organs, hepatic melanoma metastasis was not directly affected in two melanoma models. However, the loss of *Stab1* promoted effects on the local microenvironment, such as immune cell infiltration and deposition of Stabilin ligands. This study suggests that the targeting of Stab1 should be considered in a tumor- and organ-specific manner.

## Data Availability

Data are contained within the article and Supplementary Materials.

## Supplementary Materials

The following supporting information can be downloaded at: https://www.mdpi.com/article/10.3390/cancers16020441/s1, Figure S1: Body weight, weight of liver and lungs after injection with Wt31 or B16F10 *luc2.* (A–C) On day 19 after i.v. injection of Wt31 melanoma cells mice were sacrificed. Body weight (A), weight of liver (B) or lungs (C) were measured. Comparisons between Ctrl and Stab1 KO are presented. Body weight: *p* = 0.0484; liver weight: *p* = 0.1777; weight of lungs: *p* = 0.7418 (Mann–Whitney tests). (D–F) On day 14 after i.s. injection of B16F10 *luc2* melanoma cells mice were sacrificed. Body weight (D), weight of liver (E) or lungs (F) were measured. Comparisons between Ctrl and Stab1 KO are presented. Body weight: *p* = 0.5573; liver weight: *p* = 0.3144; weight of lungs: *p* = 0.6908 (Mann–Whitney tests). Figure S2: Histologic analysis of livers of Ctrl and Stab1 KO after i.v. injection with Wt31. After i.v. injection of Wt31 melanoma cells mice were sacrificed on day 19. Livers were analyzed by routine histology stainings. H&E (A), Sirius red (B), PAS (C) and Prussian blue (D) stainings of peritumoral liver tissue of Ctrl and Stab1 KO are presented. Figure S3: Histologic analysis of livers of Ctrl and Stab1 KO after i.s. injection with B16F10 *luc2*. After i.s. injection of B16F10 *luc2* melanoma cells mice were sacrificed on day 14. Livers were analyzed by routine histology stainings. H&E (A), Sirius red (B), PAS (C) and Prussian blue (Fe) (D) stainings of peritumoral liver tissue of Ctrl and Stab1 KO are presented. Figure S4: Sirius red stainings of livers of Ctrl and Stab1 KO with metastases of Wt31 or B16F10 *luc2* melanoma; microscopic size of Wt31 or B16F10 *luc2* liver metastases. A,B. Images of Sirius red stainings of hepatic metastases of Wt31 (A) or B16F10 *luc2* melanoma (B) in Ctrl or Stab1 KO livers. Scale bars: 100 µm. C. The microscopic size in mm^2^ of liver metastases of Wt31 melanoma was measured. The mean size per animal was calculated and is compared (*p* > 0.9999). D. The microscopic size in mm^2^ of liver metastases of B16F10 *luc2* melanoma was measured. The mean size per animal was calculated and is compared (*p* > 0.0635). Data information: ns = not significant. Figure S5: Vascularization and extracellular matrix composition of Wt31 or B16F10 *luc2* metastases in livers of Ctrl and Stab1 KO. (A) Immunofluorescences for DESMIN and LAMA4 of Wt31 melanoma metastases in Ctrl and Stab1 KO. Scale bars: 100 µm. (B) Immunofluorescences for DESMIN and LAMA4 of B16F10 *luc2* melanoma metastases in Ctrl and Stab1 KO. Scale bars: 100 µm. Figure S6. Analysis of the myeloid cell composition in livers of Ctrl and Stab1 KO without metastases or with melanoma metastases of B16F10 *luc2* or Wt31. (A) In untreated livers of Ctrl or Stab1 KO CD45+Gr-1 or CD45+Ly6C positive cells were analyzed by immunofluorescences. By whole slide imaging the numbers of respective immune cells were objectively quantified. The numbers of cells were set in relation to a liver area of 1 × 10^6^ µm^2^. CD45+Gr-1: *P* = >0.9999; CD45+Ly6C: *p* = 0.1143 (Mann–Whitney tests). (B) Livers of Ctrl or Stab1 KO with Wt31 melanoma metastases were analyzed by immunofluorescences for CD45+Gr-1 or CD45+Ly6C positive cells. By whole slide imaging the numbers of respective immune cells were objectively quantified. The numbers of cells were set in relation to a liver area of 1 × 10^6^ µm^2^. CD45+Gr-1: *p* = 0.8571; CD45+Ly6C: *p* = 0.4857 (Mann–Whitney tests). C. Livers of Ctrl or Stab1 KO with B16F10 *luc2* melanoma metastases were analyzed by immunofluorescences for CD45+Gr-1 or CD45+Ly6C positive cells. By whole slide imaging the numbers of respective immune cells were objectively quantified. The numbers of cells were set in relation to a liver area of 1 × 10^6^ µm^2^. CD45+Gr-1: *p* = 0.0571; CD45+Ly6C: *p* = 0.6857 (Mann–Whitney tests). Data information: ns = not significant. Figure S7. The deposition of ligands of Stab1 was determined in untreated livers of Ctrl and Stab1 KO. (A) Immunofluorescence for TGFBI, POSTN or RELN in livers of untreated, healthy 14-week-old Ctrl and Stab1 KO. Scale bars: 100 µm. (B) Quantifications of immunofluorescence (see A). The fluorescent area per 100.000 µm^2^ tissue [%] was calculated and compared between the groups. TGFBI: *p* = 0.0159; POSTN: *p* = 0.0079; RELN: *p* > 0.9999. Data information: * *p* < 0.05; ** *p* < 0.01; ns = not significant Figure S8. Stabilin ligand deposition was analyzed in livers of Ctrl and Stab1 KO with hepatic metastases of Wt31. (A) Immunofluorescence for TGFBI, POSTN or RELN in peritumoral liver tissue of Ctrl and Stab1 KO. Scale bars: 100 µm. (B) Immunofluorescence for TGFBI, POSTN or RELN in metastatic tissue of Ctrl and Stab1 KO. Scale bars: 100 µm. Figure S9. Stabilin ligand deposition was analyzed in livers of Ctrl and Stab1 KO with hepatic metastases of B16F10 *luc2*. (A) Immunofluorescence for TGFBI, POSTN or RELN in peritumoral liver tissue of Ctrl and Stab1 KO. Scale bars: 100 µm. (B) Immunofluorescence for TGFBI, POSTN or RELN in metastatic tissue of Ctrl and Stab1 KO. Scale bars: 100 µm. Table S1: List of primers for genotyping.
