# Supplementary material for: Deficiency of Stabilin-1 in the Context of Hepatic Melanoma Metastasis
Source: Cancers (Basel). 2024 Jan 19;16(2):441. doi: 10.3390/cancers16020441 (PMC10814973; doi:10.3390/cancers16020441)
Supplement: Supplementary file 1 [file cancers-16-00441-s001.zip › cancers-2805917_supplementary_table.pdf]

Supplementary Tables

Table S1. List of primers for genotyping.

Primer	Sequence
mStab1_pcr_A1F	AGACTATGGTCTCAGTCTGGGA
mStab1_pcr_B461R	CTGCAATCACTGTCCCCACACT
mStab1_pcr_C1224R	TTATTTCATACCCGCCAGTTCTGA
