# Supplementary material for: Deficiency of Stabilin-1 in the Context of Hepatic Melanoma Metastasis
Source: Cancers (Basel). 2024 Jan 19;16(2):441. doi: 10.3390/cancers16020441 (PMC10814973; doi:10.3390/cancers16020441)
Supplement: Supplementary file 1 [file cancers-16-00441-s001.zip › cancers-2805917_supplementary_figures.pdf]

Figure S1

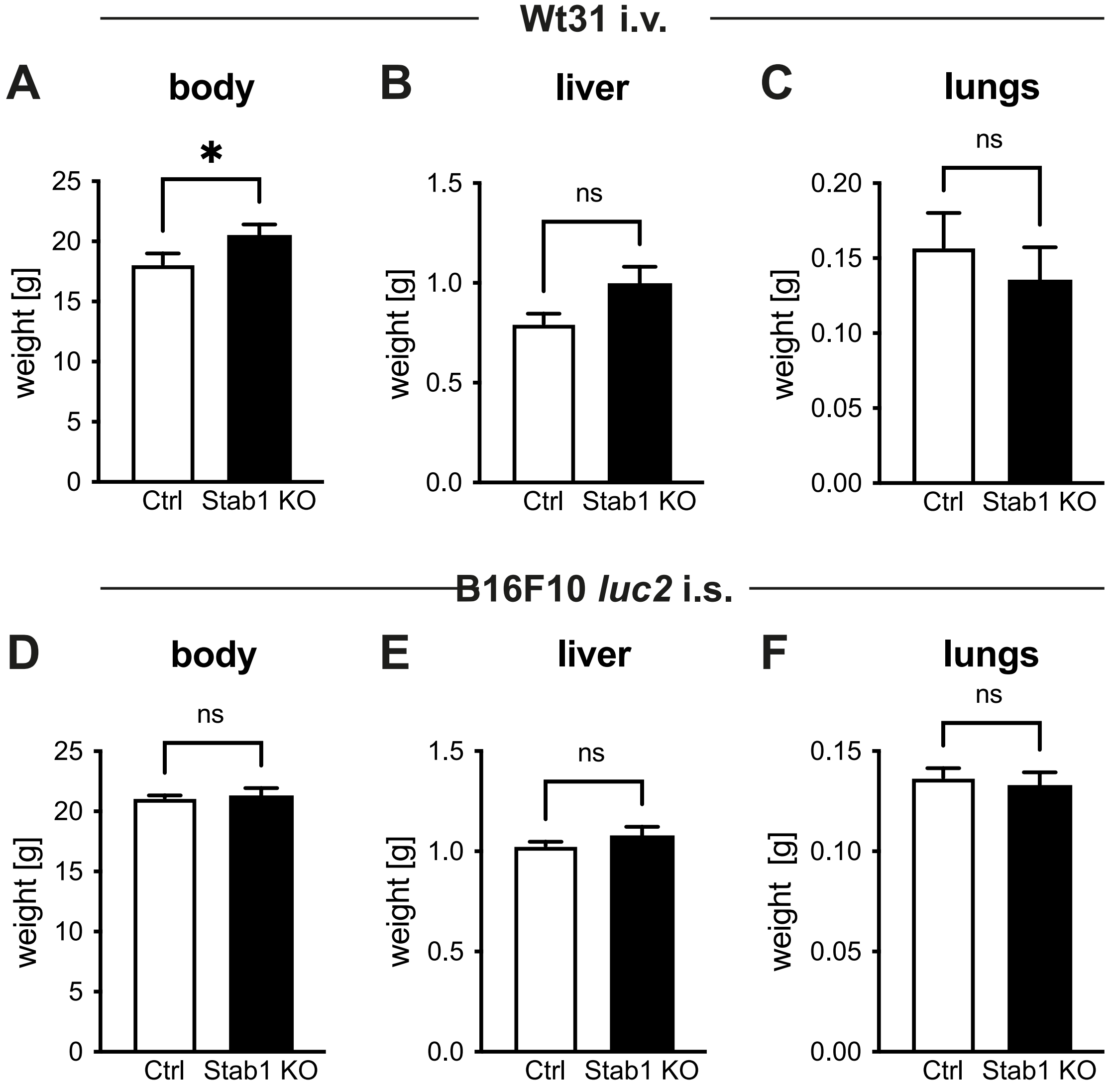

Wt31 i.v.
A
body
*
weight [g]
25
20
15
10
5
0
Ctrl
Stab1 KO
B
liver
ns
weight [g]
1.5
1.0
0.5
0.0
Ctrl
Stab1 KO
C
lungs
ns
weight [g]
0.20
0.15
0.10
0.05
0.00
Ctrl
Stab1 KO
B16F10 *luc2* i.s.
D
body
ns
weight [g]
25
20
15
10
5
0
Ctrl
Stab1 KO
E
liver
ns
weight [g]
1.5
1.0
0.5
0.0
Ctrl
Stab1 KO
F
lungs
ns
weight [g]
0.15
0.10
0.05
0.00
Ctrl
Stab1 KO

Figure S2

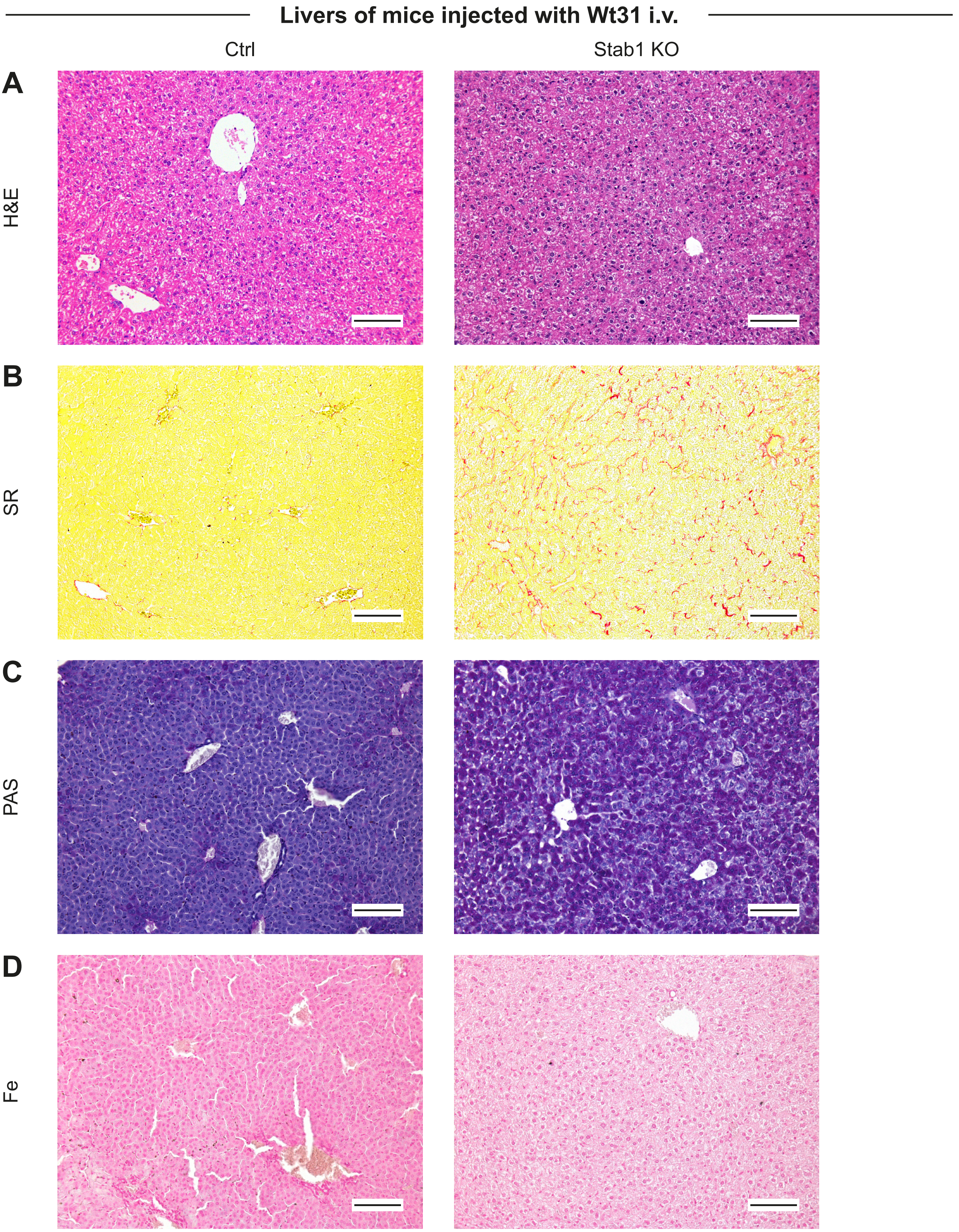
Livers of mice injected with Wt31 i.v.
Ctrl
Stab1 KO
A
H&E
B
SR
C
PAS
D
Fe

Figure S3

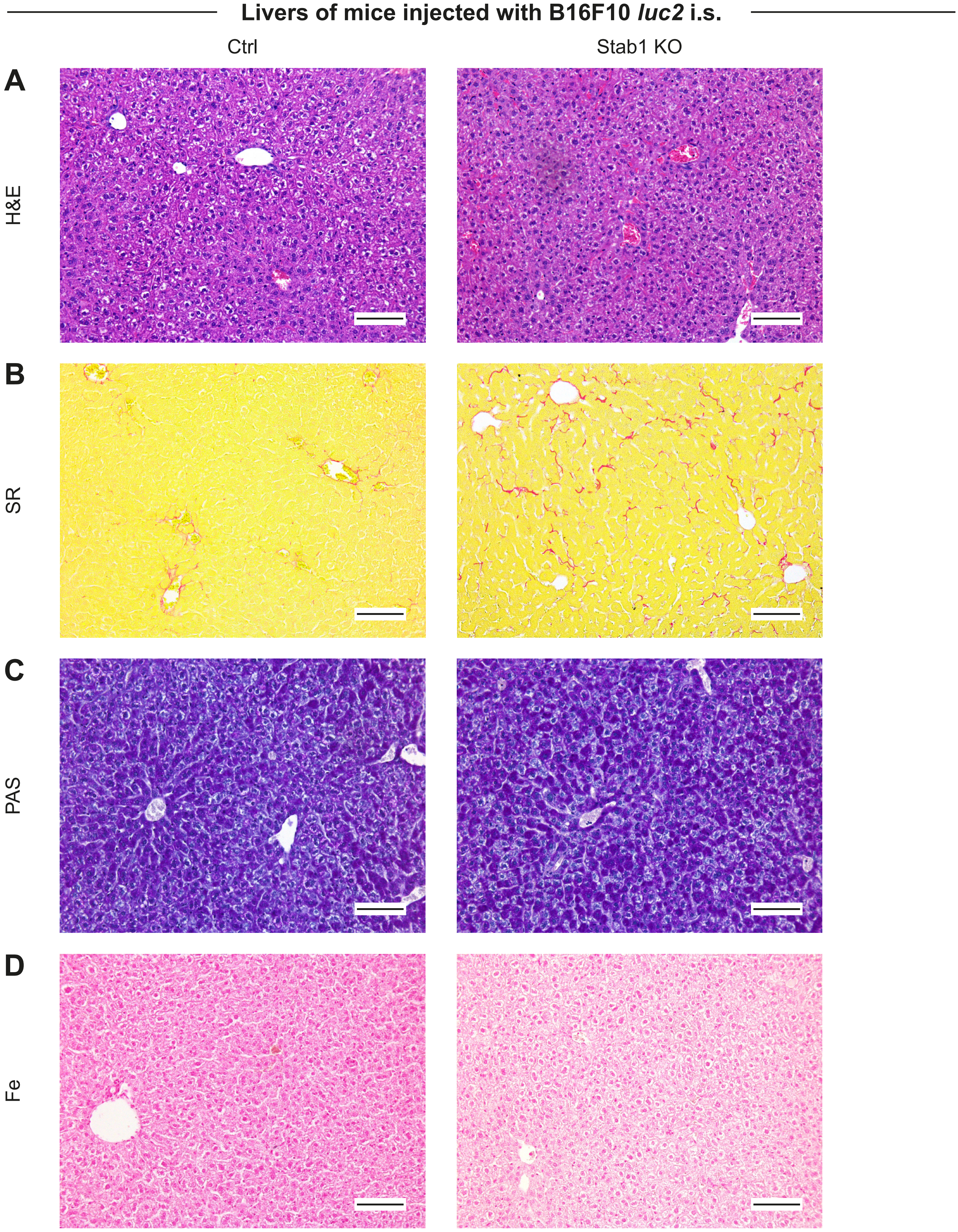
Livers of mice injected with B16F10 *luc2* i.s.
Ctrl
Stab1 KO
A
H&E
B
SR
C
PAS
D
Fe

Figure S4

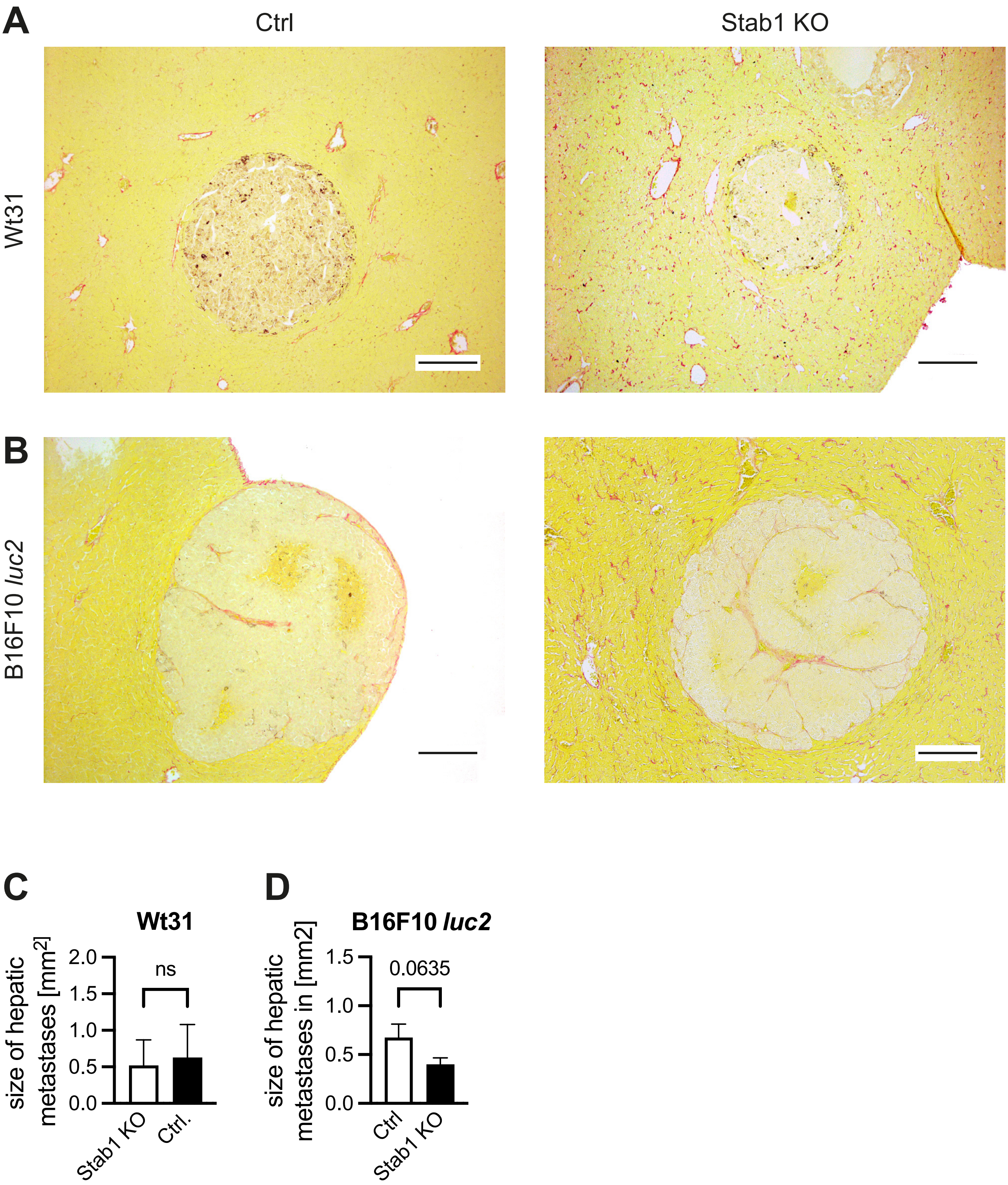

A
Ctrl
Stab1 KO
Wt31
B
B16F10 luc2
C
Wt31
size of hepatic metastases [mm2]
2.0
1.5
1.0
0.5
0.0
ns
Stab1 KO
Ctrl.
D
B16F10 luc2
size of hepatic metastases in [mm2]
1.5
1.0
0.5
0.0
0.0635
Ctrl
Stab1 KO

Figure S5

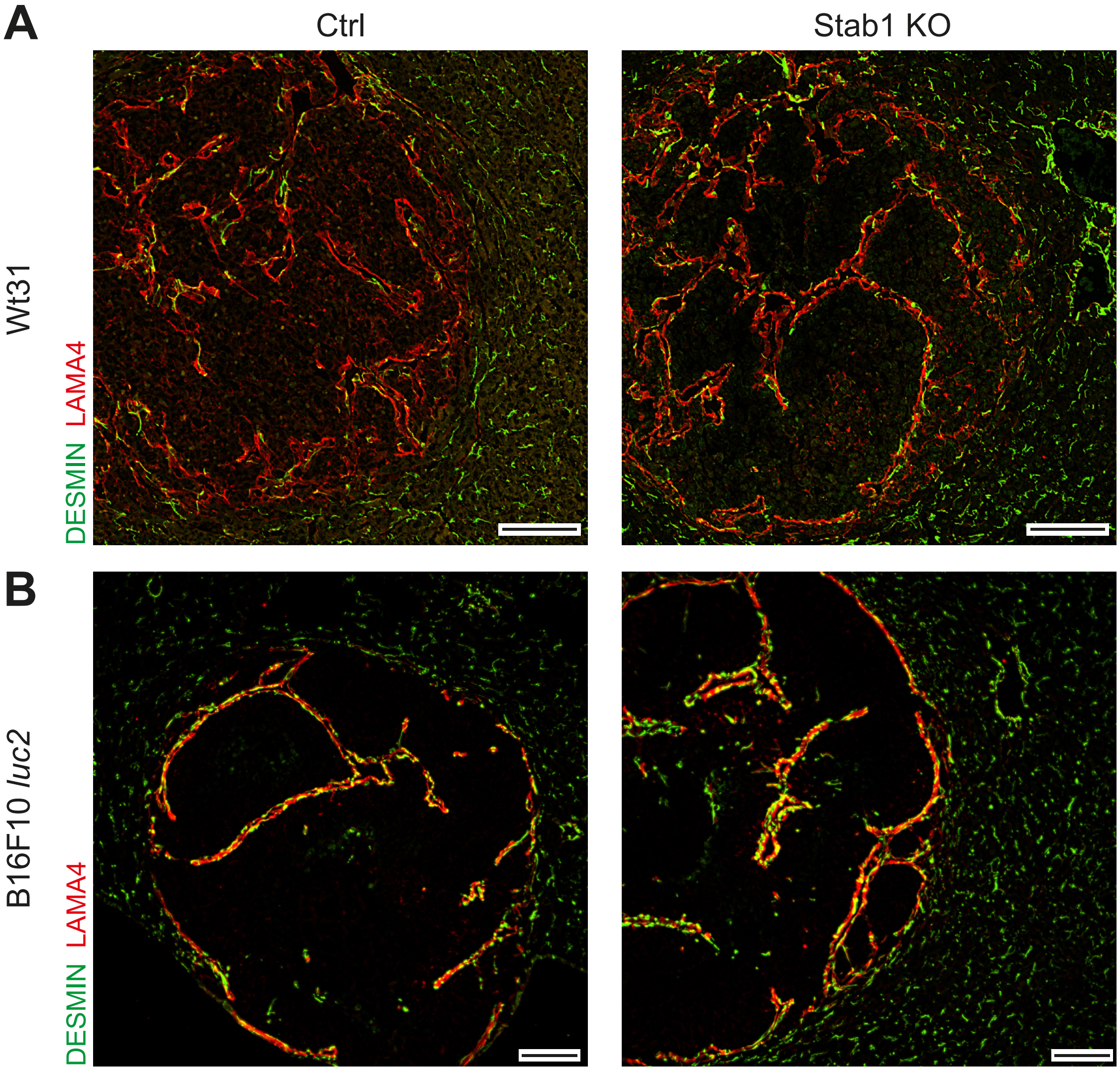

Figure S6

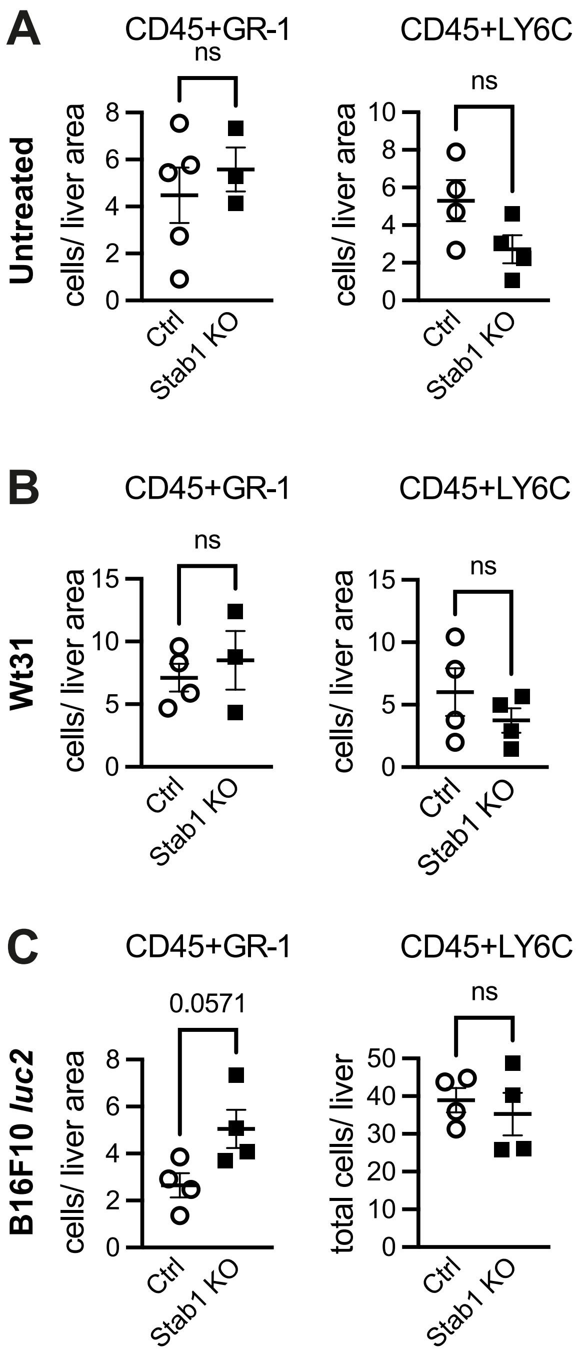
A
CD45+GR-1
CD45+LY6C
Untreated
ns
cells/ liver area
Ctrl
Stab1 KO
B
Wt31
C
B16F10 luc2
0.0571
total cells/ liver

Figure S7

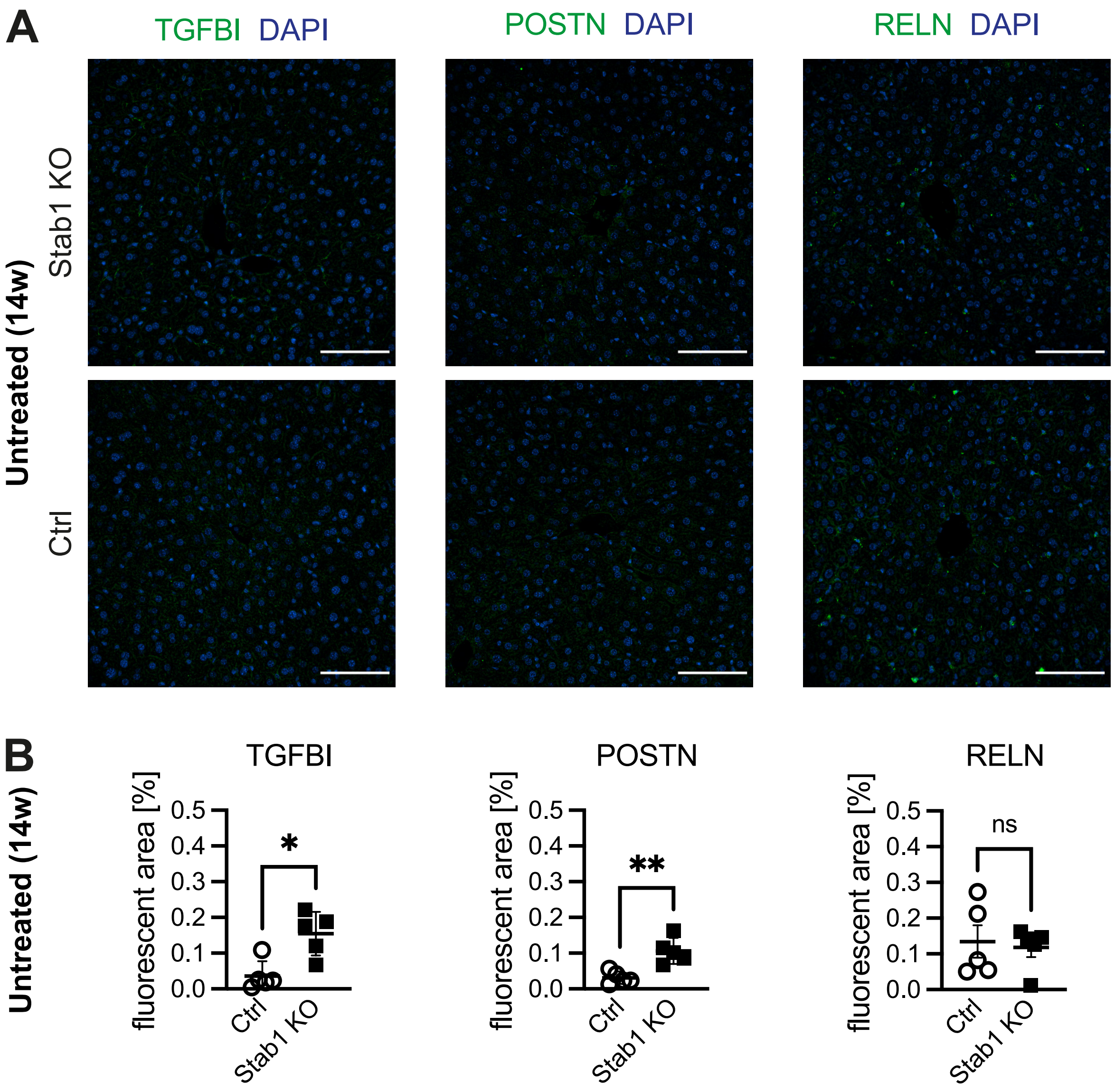
A
TGFBI DAPI
POSTN DAPI
RELN DAPI
Untreated (14w)
Stab1 KO
Ctrl
B
Untreated (14w)
TGFBI
POSTN
RELN
fluorescent area [%]
0.5
0.4
0.3
0.2
0.1
0.0
*
**
ns
Ctrl
Stab1 KO

Figure S8

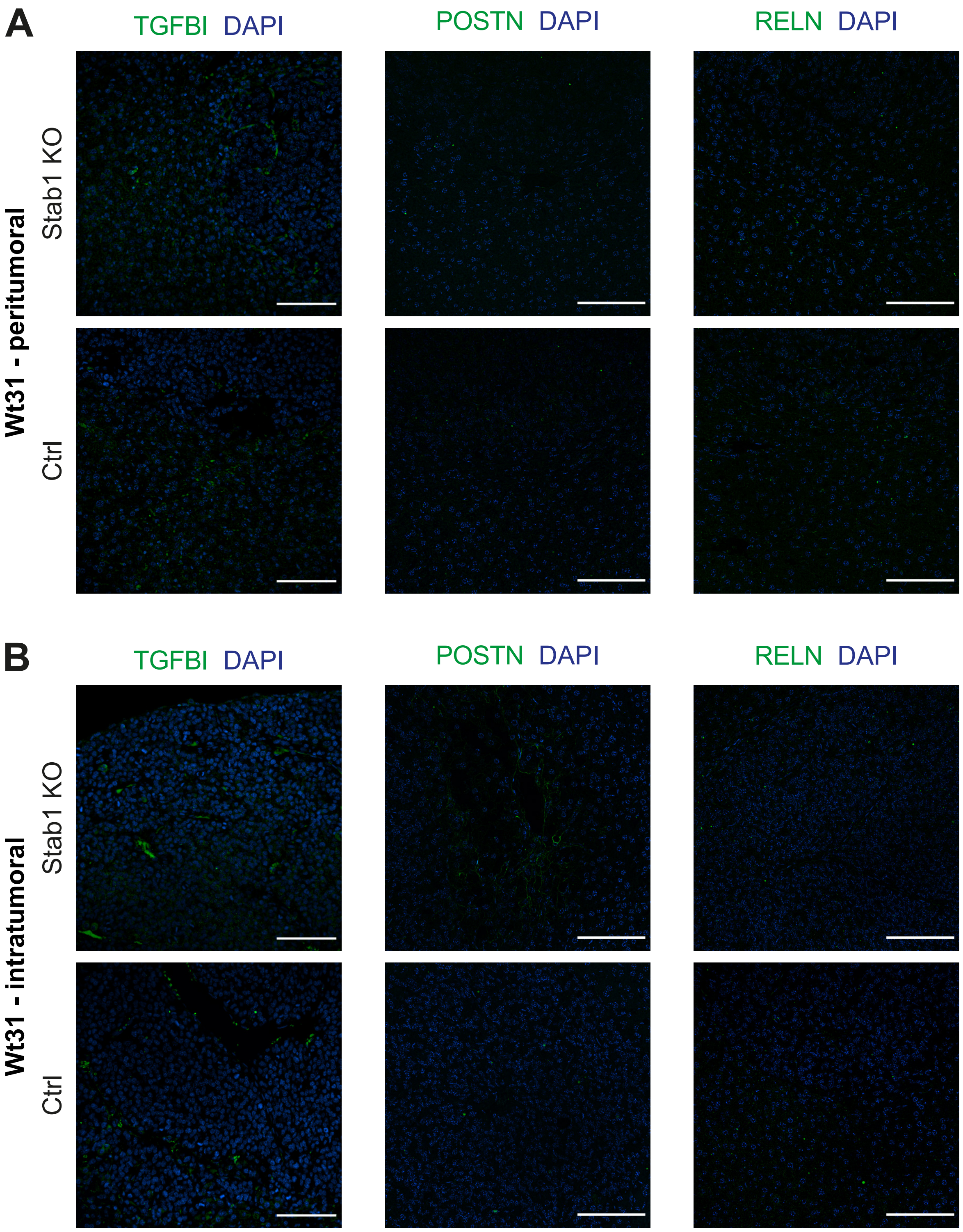
A
TGFBI DAPI
POSTN DAPI
RELN DAPI
Wt31 - peritumoral
Stab1 KO
Ctrl
B
TGFBI DAPI
POSTN DAPI
RELN DAPI
Wt31 - intratumoral
Stab1 KO
Ctrl

Figure S9

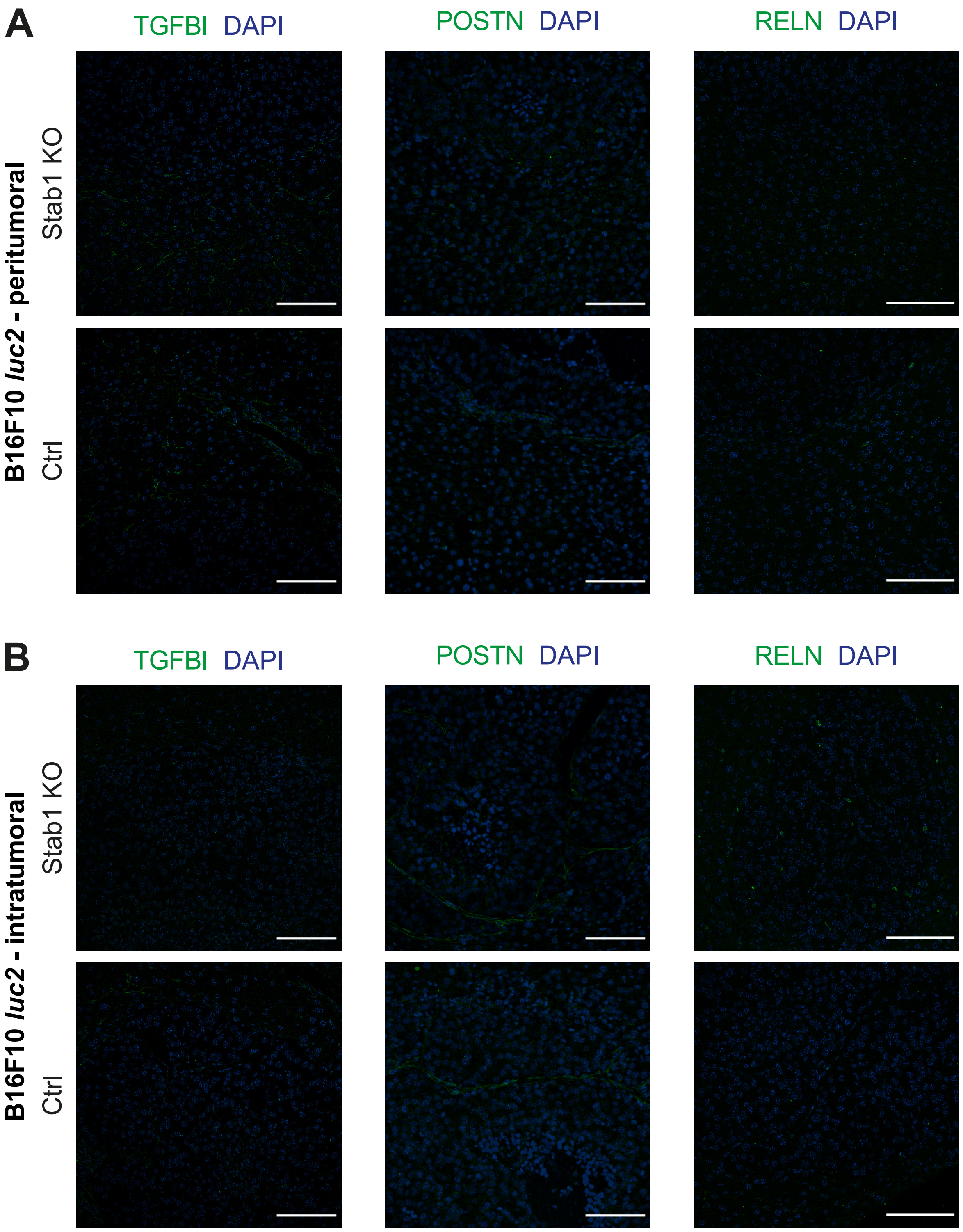
A
TGFBI DAPI
POSTN DAPI
RELN DAPI
B16F10 *luc2* - peritumoral
Stab1 KO
Ctrl
B
TGFBI DAPI
POSTN DAPI
RELN DAPI
B16F10 *luc2* - intratumoral
Stab1 KO
Ctrl
